# Family history assessment significantly enhances delivery of precision medicine in the genomics era

**DOI:** 10.1186/s13073-020-00819-1

**Published:** 2021-01-07

**Authors:** Yasmin Bylstra, Weng Khong Lim, Sylvia Kam, Koei Wan Tham, R. Ryanne Wu, Jing Xian Teo, Sonia Davila, Jyn Ling Kuan, Sock Hoai Chan, Nicolas Bertin, Cheng Xi Yang, Steve Rozen, Bin Tean Teh, Khung Keong Yeo, Stuart Alexander Cook, Saumya Shekhar Jamuar, Geoffrey S. Ginsburg, Lori A. Orlando, Patrick Tan

**Affiliations:** 1 SingHealth Duke-NUS Institute of Precision Medicine, Singapore Health Services, Singapore, Singapore; 2 Cancer and Stem Cell Biology, Duke-NUS Medical School, Singapore, Singapore; 3 SingHealth Duke-NUS Genomic Medicine Center, Singapore Health Services, Singapore, Singapore; 4 Department of Paediatrics, KK Women’s and Children’s Hospital, Singapore, Singapore; 5 Department of Physiology, National University of Singapore, Singapore, Singapore; 6 Center for Applied Genomics and Precision Medicine, Department of Medicine, Duke University School of Medicine, Durham, NC USA; 7 Cardiovascular and Metabolic Disorders, Duke-NUS Medical School, Singapore, Singapore; 8 Cancer Genetics Service, Division of Medical Oncology, National Cancer Centre Singapore, Singapore, Singapore; 9 Centre for Big Data and Integrative Genomics, Genome Institute of Singapore, Agency for Science Technology and Research, Singapore, Singapore; 10 National Heart Research Institute Singapore, National Heart Centre Singapore, Singapore, Singapore; 11 National Cancer Centre Singapore, Singapore, Singapore; 12 Department of Cardiology, National Heart Centre Singapore, Singapore, Singapore; 13 Paediatric Academic Clinical Programme, Duke-NUS Medical School, Singapore, Singapore; 14 Genome Institute of Singapore, Agency for Science Technology and Research, Singapore, Singapore

**Keywords:** Population genomics screening, Family history, Clinically actionable variants, Cancer

## Abstract

**Background:**

Family history has traditionally been an essential part of clinical care to assess health risks. However, declining sequencing costs have precipitated a shift towards genomics-first approaches in population screening programs rendering the value of family history unknown. We evaluated the utility of incorporating family history information for genomic sequencing selection.

**Methods:**

To ascertain the relationship between family histories on such population-level initiatives, we analysed whole genome sequences of 1750 research participants with no known pre-existing conditions, of which half received comprehensive family history assessment of up to four generations, focusing on 95 cancer genes.

**Results:**

Amongst the 1750 participants, 866 (49.5%) had high-quality standardised family history available. Within this group, 73 (8.4%) participants had an increased family history risk of cancer (increased FH risk cohort) and 1 in 7 participants (*n* = 10/73) carried a clinically actionable variant inferring a sixfold increase compared with 1 in 47 participants (*n* = 17/793) assessed at average family history cancer risk (average FH risk cohort) (*p* = 0.00001) and a sevenfold increase compared to 1 in 52 participants (*n* = 17/884) where family history was not available (FH not available cohort) (*p* = 0.00001). The enrichment was further pronounced (up to 18-fold) when assessing only the 25 cancer genes in the American College of Medical Genetics (ACMG) Secondary Findings (SF) genes. Furthermore, 63 (7.3%) participants had an increased family history cancer risk in the absence of an apparent clinically actionable variant.

**Conclusions:**

These findings demonstrate that the collection and analysis of comprehensive family history and genomic data are complementary and in combination can prioritise individuals for genomic analysis. Thus, family history remains a critical component of health risk assessment, providing important actionable data when implementing genomics screening programs.

**Trial registration:**

ClinicalTrials.gov NCT02791152. Retrospectively registered on May 31, 2016.

**Supplementary Information:**

The online version contains supplementary material available at 10.1186/s13073-020-00819-1.

## Background

Genetic diagnoses can inform why disease occurred, potential risks of developing further disease, and medical interventions to reduce disease development or progression [1]. Historically, family history has been used to guide risk assessment of underlying genetic predispositions in conjunction with a personal history of a medical condition. Although family history is a significant indicator for health evaluation, its collection and interpretation can be labour intensive and time-consuming and therefore overlooked or not done. Additional challenges can be encountered when interpreting family history information if collection is incomplete and details are non-specific, or insufficient training is provided to utilise family history information to support clinical decisions [2]. These challenges are further pronounced when collecting comprehensive family histories for large scale population studies.

More recently, technology advancements and declining costs have led to the increasingly widespread use of genomic sequencing, extending beyond diagnosis and treatment applications. As such, predicting health risks using genomic sequencing has expanded from cascade testing following the identification of a disease-causing variant within a family to analysing a pre-defined set of genes for a larger population. Several screening programs globally have implemented genomic sequencing for healthy or unselected populations as a first approach, irrespective of health status or family history [3–8]. These programs are exploring the potential for high quality genomic sequencing data to be integrated into healthcare delivery systems to improve health outcomes [9, 10].

In the advent of genome sequencing approaches for large populations as an initial screen, there is less emphasis on family history to identify individuals at increased risk of developing medical conditions [11]. Furthermore, there is emerging evidence from some screening programs that in unselected populations between 48 and 75% of individuals carrying a clinically actionable variant have no associated family history [4, 8, 12]. These studies suggest that genetic testing should potentially be considered in both affected and unaffected individuals, with and without an associated increased risk family history. However, whilst they suggest that family history is not an optimal tool to detect medically significant genomic variants, family history is frequently assessed only after the detection of a clinically actionable variant and therefore a direct comparison of genomic variant analysis and family history for the detection of clinically actionable variants cannot be inferred. Furthermore, some studies use electronic medical records to capture family history which has been found to be an insufficient source for medical assessment due to the limited quality information collected and recorded [1, 13, 14].

The value of family history assessment in relation to genomic screening in an unselected population is currently unknown, and therefore, it is critical to define its role in (1) identifying individuals who may benefit the most from genomic screening, (2) updating current understanding of variants of uncertain significance (VUS), (3) suggesting the presence of clinically actionable variants in genes undiscovered or unknown to be associated with disease, and (4) indicating the possibility of a protective gene-gene or gene-environment interaction. To complement existing genomic screening programs, we conducted for the first time, a comprehensive assessment of high-quality family history alongside genomic data by systematically collecting at least a three generation family history at the time of genomic sequencing. We compared the detection of clinically actionable genomic variants in 95 cancer predisposition genes amongst 1750 participants with no known pre-existing medical conditions according to family history availability and risk assessment by family history.

## Methods

### Study design and participants

This cohort study conducted in Singapore was an exploratory analysis of the relationship between variant status on genome sequencing (clinically actionable or not) and cancer risk level (increased or average) based on family and medical history of unselected healthy Singaporeans. The participants were recruited for a prospective institutional review board-approved Biobank (SingHealth Central Institutional Review Board in 2014) or SingHEART study (https://clinicaltrials.gov/ct2/show/study/NCT02791152, retrospectively registered on May 31, 2016) conducted at the National Heart Centre Singapore between August 2014 and December 2018 [15]. Details of participant recruitment and methods of both Biobank and SingHEART studies have been previously described [15, 16], and the inclusion/exclusion criteria can be found in Table 1. Briefly, volunteers with no known pre-existing health conditions over 16 years of age were recruited in response to a research advertisement in the local paper in 2014. They consented to a detailed medical screen and a genetic screen using whole genome sequencing (WGS) technology. MeTree (an online family history collection tool) [17] was incorporated into the recruitment process in 2016 to systematically collect family history. MeTree has been shown to increase the quality of family history data provided by patients [18]. Prior to the incorporation of MeTree, family history was not collected at recruitment. All participants included in this study were asymptomatic as ascertained by their health screen at recruitment and none reported a previous diagnosis of cancer.

**Table 1:** Inclusion/exclusion criteria for Biobank and SingHEART research studies

**Inclusion/exclusion criteria for Biobank**	
Inclusion criteria:	
1. Healthy men and women age ≥ 16 years and ≤ 90 years	
Exclusion criteria:	
1. History of heart attack 2. Percutaneous coronary intervention (PCI)—a procedure used to open blocked coronary arteries (caused by coronary artery disease) 3. Coronary heart disease with stenosis (abnormal narrowing of the blood vessel) of more than 75% 4. History of stroke 5. Currently being pregnant 6. Known definite diabetes mellitus or on treatment for diabetes mellitus 7. Cardiac pacemaker, brain aneurysm or clips, electronic implant or prosthesis (artificial body part such as leg), eye metal foreign body injury, or severe claustrophobia (fear of small or confined places) 8. Medication which **does not** include 1 anti-hypertension medication, oral contraceptive pill, asthma inhalers, nonsteroidal anti-inflammatory drugs (NSAIDs) 9. Parents, children, or siblings (first degree family members) that have an inherited heart condition—either hypertrophic cardiomyopathy (HCM) or dilated cardiomyopathy (DCM) 10. Family members already volunteered for this study—parents, children, siblings, grandparents, great grandparents, cousins, nieces, or nephews	
**Inclusion/exclusion criteria for SingHEART**	
Inclusion criteria:	
1. Healthy men and women age 21–69 years	
Exclusion criteria:	
1. Previous myocardial infarction (MI). This will include ST-elevation MI (STEMI) and non-ST-elevation MI (NSTEMI) 2. Known coronary artery disease—prior coronary revascularization 3. Known documented peripheral arterial disease 4. Previous stroke (stroke is defined as new focal neurological deficit persisting more than 24 h) 5. More than ongoing use of 2 or more anti-hypertensive agents 6. Prior history of cancer (excludes pre-cancerous lesions) 7. Expected life expectancy less than 1 year 8. Known definite diabetes mellitus or on treatment for diabetes mellitus 9. Known autoimmune disease or genetic disease 10. Known endocrine disease on treatment 11. Psychiatric illness 12. Asthma or chronic lung disease requiring long term medications or oxygen 13. Chronic infective disease, including tuberculosis, hepatitis B and C, and HIV 14. Inability to comply with study protocol 15. Any other acute or chronic medical or physical condition deemed by the investigator to affect study outcomes.	

### Family history collection

For participants recruited after incorporating family history collection into the study protocol, participants were notified prior to their initial recruitment appointment to gather medical information from their family members. Some cultural differences were observed when family history collection commenced, in particular to how relationships were viewed, as outlined in Bylstra et al. [16]. At their recruitment appointment, family history was collected using MeTree which collects up to four generations of family health information extending from children to grandparents and cousins. MeTree provides selection for over 20 cancer types and syndromes with explanations about different types of cancers and how to distinguish primary from secondary tumour sites. It also prompts for a range of other conditions, such as heart disease and diabetes, and has the ability to enter free text so that any cancer or medical condition occurring in a family can be captured [19]. Current US clinical guidelines are incorporated into the generation of personalised risk reports for patients and their providers [20]. By providing online instructions about how to collect family history and what information should be reported, the information captured by MeTree has previously been shown to be sufficient in performing risk assessments on the majority of patients [18]. This is consistent with other independent studies validating the improved quality and content of family history collection using online collection tools [21, 22].

### Risk assessment based on family history

Each family history documenting a presence of cancer was assessed by the clinical genetics team in accordance with clinical testing criteria guidelines, National Comprehensive Cancer Network (NCCN) Genetic/Familial High-Risk Assessment: Breast and Ovarian (Version 3.2019) [23], and Genetic/Familial High-Risk Assessment: Colorectal (Version1.2018) [24] or supplemented by an organ-specific international guideline to determine the risk of developing cancer [25, 26]. In cases where the familial risk was unclear because of incomplete information pertaining to cancer type, age of diagnosis, or disease progression, the family pedigree was reviewed in further detail by the clinical genetics team, taking into consideration participant age and number of family members until a consensus of risk was reached.

### Cancer genes for analysis

A list of 95 genes associated with tumour and cancer development was devised from genes studied in the literature and/or published gene lists [27–29] or commercially available gene panels such as Illumina TruSight Cancer gene panel and WuXi NEXTCode cancer gene panel available through clinical sequence analyzer (www.genuitysci.com/products/clinical-sequence-analyzer). This gene list was subsequently compared with databases such as Online Mendelian Inheritance in Man (OMIM) [30] to clarify cancer associations and Clinical Genome Resource (ClinGen) [31] for evidence of disease validity. There are 25 genes associated with a cancer phenotype in the American College of Medical Genetics and Genomics 59 secondary findings gene list (ACMG SF v2.0) [32], and these were included in our 95 cancer gene panel. The gene list was reviewed and refined by clinical experts. The resulting 95 genes and their disease association according to ClinGen and OMIM can be found in Additional file 1: Table S1.

### Genomic sequencing and classification

DNA was extracted from a donated blood sample and WGS was performed with a third party provider using the Illumina HiSeq X platform under standard protocols. Data was returned in the form of FASTQ files and analysed using an in-house bioinformatics pipeline as previously described [16].

Variants occurring in the customised cancer gene panel were filtered by frequency against our local population-matched database comprising of 3500 exomes and gnomAD v3 (https://gnomad.broadinstitute.org/).

#### Likely pathogenic/pathogenic variant classification

Variants for further review were selected according to one of the following criteria:

1. At least one entry classification as “likely pathogenic” or “pathogenic” in ClinVar [33] using VCF release 20190408 for GRCh37, in both exonic and intronic regions
2. Variants that cause disruption to protein function (small insertions, small deletions, stopgain, stoploss, or disruption of an essential splice site) and minor allele frequency (MAF) of < 1%. Haploinsufficiency for each gene was assessed by reviewing literature and recommendations from ClinGen (accessed until May 2019).
3. Variants absent in ClinVar, a MAF of < 1% and high in silico prediction (REVEL > 0.7) [34]

Allele frequency, in silico prediction, literature (PubMed, Human Gene Mutation Database (HGMD) [35], Google Scholar, and LitVar [36] were assessed and classified according to the American College of Medical Genetics and Genomics (ACMG) and the Association for Molecular Pathology (AMP) variant classification criteria guidelines [37]. Consensus for the variant classification was obtained by discussion amongst genetics specialists. For each variant classified as either likely pathogenic or pathogenic, the QC metrics and corresponding BAM files were then visually inspected for confirmation. For variants where the QC metrics and/or presence in BAM was ambiguous, these were then validated by Sanger sequencing.

#### Variants of uncertain significance selection

The total number of variants of uncertain significance (VUS) was selected by their classification in InterVar as VUS and MAF of < 1%. Those with potential pathogenicity were selected by a high in silico prediction (REVEL > 0.7), rare in the population (MAF < 1% and either absent in ClinVar or present in ClinVar as VUS (VCF release 20190408 for GRCh37). Supporting literature, if available, was reviewed and variants were classified according to ACMG-AMP criteria. A flowchart of the variation curation process can be found in Additional file 1: Fig. S1.

Family history was not taken into consideration for the variant classification process. Once variant classification was established, corresponding family history, if available, was examined and then allocated to one of the three comparison cohorts.

### Statistical analysis

Relative risk (RR) was calculated as specified by Altman et al. 1991 [38]. All statistical tests were two-tailed, and a *p* value less than 0.05 was considered statistically significant.

## Results

### Baseline characteristics

Over the 4 year study period, we recruited 1750 participants (Table 2), with a median age of 45 years (range 16–88 years of age). Fifty-two percent of participants were females, with a slight over-representation of individuals of Chinese ethnicity (89.3% vs 74.3% in the general population of Singapore). Family history (FH) was available for 866 (49.4%) participants. There was no difference in baseline characteristics for age and gender between the cohorts with and without family history (FH available and FH unavailable cohorts) (Table 2). However, there were fewer individuals of Malay ethnicity in the FH available cohort (*p* value = 0.017).

**Table 2:** Baseline characteristics

	Overall total (***n*** = 1750)	FH available (***n*** = 866)			FH not available (***n*** = 884)
		*Increased FH risk (n = 73)*	*Average FH risk (n = 793)*	***Total (n = 866)***	
Median age, in years (range)	45 (16–88)	45 (21–72)	44 (19–77)	**45 (19–77)**	**43 (16–88)**
Gender, female (%)	912 (52.1)	47 (64.4)	403 (50.9)	**450 (52.0)**	**462 (54.7)**
Ethnicity					
Chinese (%)	1562 (89.2)	65 (90.3)	714 (90.1)	**779 (89.9)**	**783 (88.3)**
Indian (%)	75 (4.2)	5 (5.6)	31 (3.9)	**36 (4.2)**	**39 (.44)**
Malay (%)	68 (3.8)	1 (1.4)	23 (2.9)	**24 (2.8)**	**44 (4.9)**
Other (%)	45 (2.6)	2 (2.8)	25 (3.1)	**27 (3.1)**	**18 (2.0)**

### Family history

Amongst the 866 participants where family history was available, 73 (8.4%) were identified as having increased risk of developing cancer (increased FH risk cohort) based on clinical testing guidelines, whilst the remaining 793 (91.6%) were considered to not have an increased risk of developing cancer (average FH risk cohort). All baseline characteristics were similar between the two cohorts with the exception of slightly more females in the increased FH risk cohort (*p* value = 0.026), which could be attributed to the prevalence of breast cancer syndromes (Table 2).

An overview of the cancers reported in each family in the increased FH risk cohort is provided in Additional file 1: Fig. S2. Breast cancer (38.3%) was the most common, followed by ovarian (17.8%) and colorectal cancer (10.9%). Some participants indicated multiple family members with early-onset cancer, e.g. aged 30s and 40s but were unaware of the cancer type, forming the unknown category (10.9%). More than one cancer type was reported in 57% of families.

### Genome sequencing

**Fig. 1:**
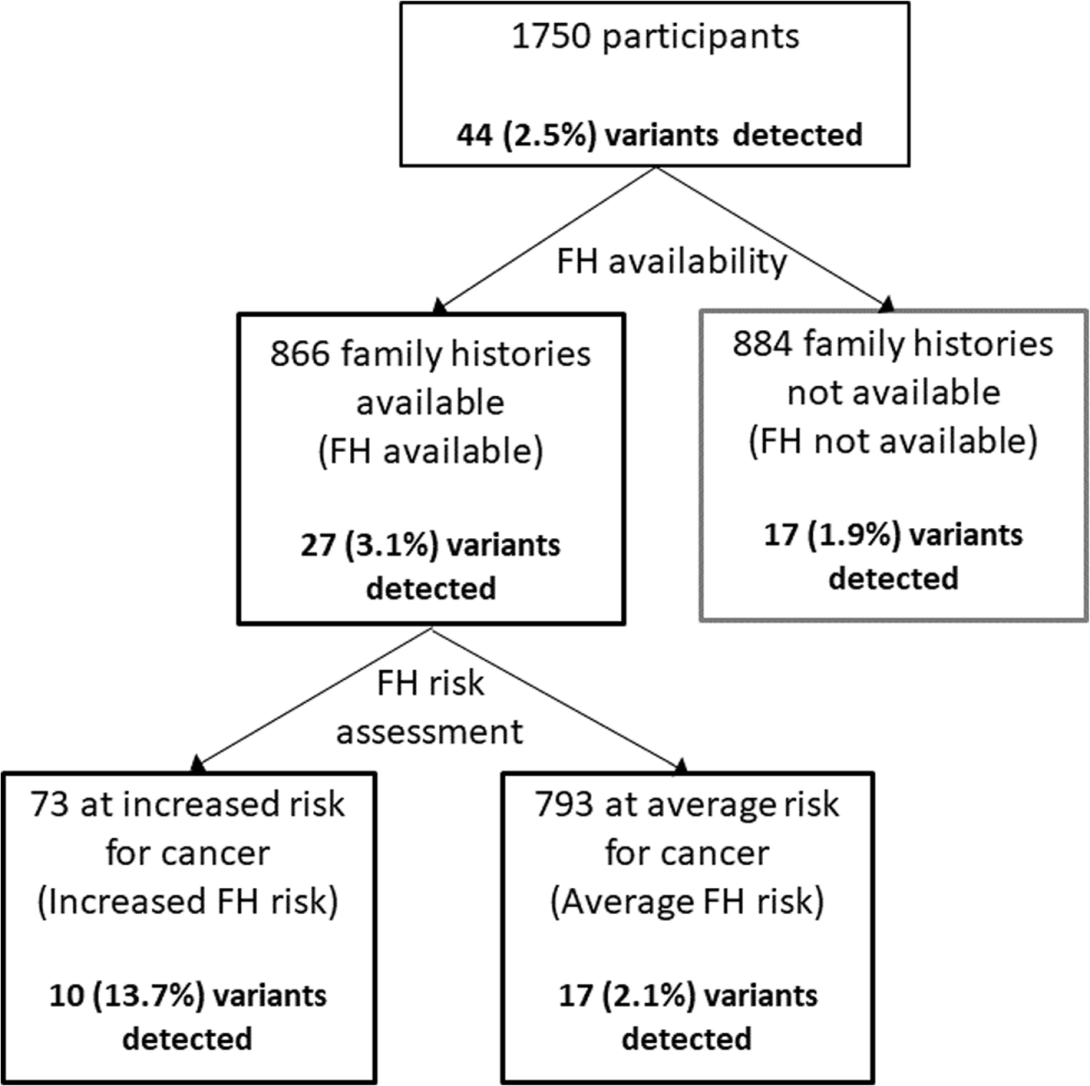
Family history assessment and clinically actionable variant detection overview

We performed genome sequencing on all 1750 participants and analysed the genomic data for clinically actionable variants in the target genes. We identified a total of 4937 rare variants across the 1750 participants in the 95 target genes with a MAF of < 1%. We identified 44 pathogenic or likely pathogenic variants (Fig. 1) and 2632 VUS in the 95 target genes which were identified using InterVar [39] VUS classification. For the purpose of this analysis, we focused on the pathogenic or likely pathogenic variants, henceforth referred to as clinically actionable variants. No participants had more than one clinically actionable variant or were found to be homozygous or compound heterozygous for an autosomal recessive condition. Furthermore, no participants were carriers of an autosomal recessive condition which was present in their family.

### Comparison of genomic variants between cohorts

Overall, 44 clinically actionable variants (2.5%) were detected amongst the total cohort of 1750 participants. We compared the frequency of clinically actionable variant amongst the cohorts. The number of clinically actionable variants detected between the FH available cohorts (27 variants) and FH not available cohort (17 variants) was not statistically significant (3.1% vs 1.9%, *p* = 0.158). However, once ascertained for family history risk, the variants with clinical significance were more frequent in the increased FH risk cohort (10 variants) compared to the average FH risk (17 variants) and FH not available cohorts (17 variants) (Fig. 1). Amongst the increased FH risk cohort, 13.7% (1 in 7) unrelated participants were found to have a clinically actionable variant in comparison to 2.1% (1 in 47) of unrelated participants in the average FH risk cohort (*p* = 0.00001) and 1.9% (1 in 49) of participants in the FH not available cohort (*p* = 0.00001).

When focusing on the 25 cancer genes in the ACMG SF v2.0 gene list, there was an even higher chance of detecting a clinically actionable variant in the increased FH risk cohort (1 in 14 or 6.8%) compared to the average FH risk cohort (1 in 264 or 0.4%) or FH not available cohort (1 in 211 or 0.5%) (Table 3).

**Table 3:** Comparison of clinically actionable variants identified in FH not available and available cohorts

		Increased FH risk ***n*** = 73	Average FH risk ***n*** = 793	RR (95% CI)	***p*** value	Increased FH risk ***n*** = 73	FH not available ***n*** = 884	RR (95% CI)	***p*** value
**Cancer gene panel**									
No of clinically actionable variants (%)		10 (13.7)	17 (2.1)	6.39 (3.0–13.4)	0.0001	10 (13.7)	17 (1.9)	7.1 (3.3–14.9)	0.0001
**ACMG SF v2.0 cancer genes**									
No of clinically actionable variants (%)		5 (6.8)	3 (0.4)	18.1 (4.4–74.2)	0.0002	5 (6.8)	4 (0.5)	15.13 (4.1–55.1)	0.0001

*RR* relative risk, *CI* confidence interval

Increased FH risk: participants assessed at increased cancer risk based on their family history

Average FH risk: participants who were not found to be at increased risk based on their family history

FH not available: participants where family history was not available

### Relationship between genomic variants and family history

Amongst the FH unavailable cohort, 17 clinically actionable variants were found in 13 cancer genes; four of these occurred in three genes from the ACMG SF v2.0 gene list (Additional file 1: Table S2 and S3).

Focusing on the 866 participants where family history was available, 73 (8.4%) participants had increased risk family history and 27 (3.1%) participants had a clinically actionable variant in one of the 95 target genes (Table 4). There were 786 participants (90.7%) where the family history risk and clinically actionable variants were concordant. Out of this, as expected, 776 (89.6%) participants who were in the average risk cohort did not carry any clinically actionable variants.

**Table 4:** Relationship of clinically actionable variants and family history within the FH available cohort (*n* = 866)

	Family history risk, ***n*** (%)		
	Increased	Average	Total
**Clinically actionable variants,** ***n*** **(%)**			
**Present**	10 (1.2)	17 (2.0)	27
**Absent**	63 (7.3)	776 (89.6)	839
**Total**	73	793	**866**

#### Concordant cases—increased FH risk and clinically actionable variant detected

Ten (1.2%) participants were at increased risk ascertained by both genomic analysis and family history. Of the ten clinically actionable variants detected, five of these were found in three of the genes in the ACMG SF v2.0 gene list (Additional file 1: Table S2). Nine participants carried a clinically actionable variant where the association with their family history was well established. However, there was one participant with a likely pathogenic *AXIN2* variant and a family history of breast cancer—clinical evidence regarding this association is only emerging (Additional file 1: Table S4).

#### Discordant cases—clinically actionable variant detected with average FH risk

There were 80 (9.2%) participants of the FH available cohort where the family history risk and clinically actionable variants were discordant. Of these, 17 (2.0%) were found to carry clinically actionable variants yet they were at average risk according to their family history. Seventeen clinically actionable variants were found in 11 cancer genes. Nine of these participants reported a family history of cancer, seven were not associated to the clinically variant detected, and two were associated; however, as the age of diagnosis was older or unknown, they did not meet the pre-specified clinical testing criteria for increased risk. Three clinically actionable variants occurred in *BRCA2*, a gene included in the ACMG SF v2.0 gene list, yet only one of these participants had a corresponding family history of cancer (a grandmother diagnosed with breast cancer in her 60s) and the remaining were found in lower penetrant genes or genes where evidence associated with cancer development is still emerging (Table 5).

**Table 5:** Clinically actionable variants and associated family history in the average FH risk cohort

Average FH risk		
**176**	*ATM* c.8545C>T p.R2849X*	Father d. heart failure 80y, sister dx breast cancer, brother dx hypertension
**67**	*ATM* c.8435_8436del p.S2812fs*	Father dx hypertension 60s, high cholesterol and heart attack d.70s, lung disease 80s, mother dx hypertension 50s and high cholesterol 60s, maternal grandmother dx colorectal cancer and hypertension 50s, maternal grandmother d. lung disease 80s, paternal grandmother dx lung disease d.60s
**53**	*BLM* c.1291_1292InsTCAGGCCTCCATAGA	Mother dx slight stroke 70s d.85y, daughter dx thyroid issues 25y
**15**	*BRCA2* c.9684delT p.S3229fs*	Father dx high blood sugar 58y, paternal grandfather dx. lung cancer 50s d.50s, paternal aunt dx diabetes 50s, paternal uncle dx colorectal cancer/liver cancer 60s
**767**	*BRCA2* c.5576_5577del p.T1859fs*	Father dx heart disease adolescence and hypertension 60s d.60s, mother dx hypertension, diabetes and diabetic kidney disease 70s, sister dx hypertension 60s, maternal grandmother dx breast cancer 60s
**750**	*BRCA2* c.7805+3A>C	Father dx prostate issues and heart attack 60s, mother dx thyroid disease 50s and hypertension 60s, maternal grandmother d. stroke 60s, patneral grandmother d. lung disease 80s, paternal aunt dx Parkinson disease 70s
**637**	*BRIP1* c.1343G>A p.W448X	Father dx hypertension, mother dx diabetes d.55y, 2 sisters dx diabetes
**169**	*DICER1* c.1353_1360del p.R451fs*	Father dx hypertension 70s and lung cancer 80s, mother dx hypertension, obesity and rheumatoid arthritis 70s, maternal aunt 3 dx colorectal 80s d.80s, 1 paternal aunt d. stroke 60s, 1 paternal aunt dx breast cancer 60s d.60s
**2**	*GPC3* c.67C>T p.Q23X	Father dx hypertension 40s, paternal grandmother d. heart attack 60s
**76**	*LZTR1* c.1018C>T p.R340X	Mother dx high cholesterol 70s, maternal aunt dx ischaemic stroke 70s
**81**	*LZTR1* c.465C>G p.Y155X*	Father dx hypertension 40s and heart disease 60s, mother dx high cholesterol and diabetes 50, maternal grandmother dx diabetes 80s, paternal grandmother dx liver cancer 70s d.80s, paternal uncle dx hypertension 60s
**454**	*RAD50* c.2165delA p.L722fs*	Father dx COPD, mother dx cervical cancer 50s, maternal uncle dx prostate cancer 50s, paternal grandmother dx diabetes d. diabetes-related complications 80s, paternal grandfather dx unknown cancer d.90s, paternal uncle dx diabetes
**281**	*RAD51C* c.394dupA p.G132fs*	Maternal grandfather d. unknown cancer 50s, brother dx unknown liver condition 30s
**662**	*RAD51D* c.330_331insTA p.K111fs	Father dx with hypertension, mother dx with diabetes
**118**	*RAD51D* c.330_331insTA p.K111fs*	Father dx heart attack 79y, mother dx hypertension and diabetes, 1 sister dx hypertension, 1 sister dx unknown cancer 63y
**671**	*RAD51D* c.330_331insTA p.K111fs*	Father dx bladder cancer 65y and stroke 80y d.85y, mother dx transient ischemic attack 93y, brother dx hypertension and colorectal cancer 76y d.76y, sister dx fibroids and had thyroidectomy
**64**	*XRCC2* c.280dupA p.T94fs	Father dx ?lung/throat cancer 50s d.59y

dx diagnosis, d. died,* y *year

*Report of an associated cancer family history

Transcripts: ATM:NM_000051.3, AXIN2:NM_004655.4, BLM:NM_000057.4, BRCA1:NM_007300.4, BRCA2:NM_000059.3, BRIP:NM_032043.3, DICER1:NM_030621.4, GPC3:NM_001164617.2, LZTR1:NM_006767.4, MSH2:NM_000251.3, RAD50:NM_005732.4, RAD51C:NM_058216.3, RAD51D:NM_002878.3, SUFU:NM_016169.4, XRCC2:NM_005431.2

#### Discordant cases—increased FH risk but no clinically actionable variant detected

Conversely, there were 63 (7.3%) participants at increased family history risk where no clinically actionable variant was found (Table 4). As we adopted strict classification criteria to annotate the variant pathogenicity, we also considered VUS variants which could be possible candidates for pathogenicity. We found nine VUS variants amongst the 63 participants which were associated with their corresponding increased risk family history (Additional file 1: Table S5). For example, one participant was at increased risk of colorectal cancer due to an affected maternal grandmother and aunt and carried a *PMS2* p.R813W variant of unknown significance which is rare in the population and predicted to be deleterious by in silico models. With further investigation, such as segregation of these variants amongst affected individuals in the same family, it is possible that the pathogenicity of these variants may be clarified further however this was beyond the scope of this study.

## Discussion

As family history has been long understood to play a vital role in targeting underlying genetic causes, we conducted an in-depth assessment of systematic family history collection and genomic data in a population genomic screening study setting. Using this data, we were able to define overlapping health risk identifiers attributed by family history and genetic factors.

Amongst a cohort of 1750 participants who had undergone genome sequencing, 866 family histories of at least three generations were collected using validated family history software [40]. Family history assessment identified 73 participants at increased risk of developing cancer and 1 in 7 participants carried an autosomal dominant clinically actionable variant which was a sixfold increase when compared to the FH average risk cohort (1 in 47) and a sevenfold increase when compared to the FH not available cohort (1 in 52). This threshold was further pronounced when selecting for the 25 cancer genes in the ACMG SF v2.0 gene list amongst the increased FH risk (1 in 14 or 6.8%) versus the average FH (1 in 264 or 0.4%) and FH not available (1 in 221 or 0.5%) cohorts, indicating that not only were the clinically actionable variants more prevalent in the increased FH risk cohort, additionally a higher proportion of highly penetrant genes was also detected. The prevalence of clinically actionable variants in ACMG SF v2.0 cancer genes in the increased FH risk cohort was also considerably higher than other reported studies that assessed the presence of pathogenic variants in the ACMG SF v2.0 gene list of unselected populations ranging from 1.5% [41] to 2.7% [42] and 1.6% in an ethnically similar cohort [43].

By integrating a standardised and high quality family history assessment, our findings indicate that selecting participants according to their family history for genomic testing significantly increases the likelihood of detecting carriers for cancer syndromes. Therefore, the traditional triaging of participants by family risk assessment in our study appears to be an effective adjunct intervention to increase the detection of clinically actionable variants and a useful tool to frame expectations when counselling about the likelihood of detecting disease-causing variants. Following the detection of a clinically relevant variant, there is support that collection of family history in conjunction with genotype can also contribute to tailored advice regarding disease penetrance. Although not detected in this study, family history, for example, can modify the clinical management of *PALB2* clinically actionable variant carriers*. PALB2* is associated with an increased risk of breast cancer, and the absolute risk for *PALB2* carriers by 70 years of age is 33% in the absence of a breast cancer family history but up to 58% for a female carrier with two or more first-degree relatives with breast cancer diagnosed by 50 years of age [44]. Guidelines such as NCCN [23] and EviQ (eviq.org.au) acknowledge the consideration of screening versus risk reducing surgery based on the presence of a breast cancer family history. In this case, family history is valuable in providing individual disease penetrance risk and may enable a further understanding of familial risk modifiers, in particular, for genotype-phenotype correlations in unaffected populations.

These findings contrast with some recent cohort studies suggesting family history is not a useful tool for identifying carriers of monogenic conditions, as at least half of the carriers detected in their unselected populations did not present with an associated increased risk family history, nor would have met eligibility criteria for genetic testing [4, 8, 12]. We also detected carriers of cancer syndromes that did not meet testing guidelines according to their family history (17 participants), half of which had no family history of cancer. Although a proportion of these variants could be de novo, overall they were found in genes more recently understood to cause cancer with less clinical information available and lower penetrance such as *DICER1*. This was further indicated by the presence of fewer ACMG SF v2.0 cancer variants in comparison to the increased FH risk cohort. There could also be protective gene-gene and/or gene-environment interactions present that were not tested for in this study. It is also possible that the family histories are incomplete and that further relevant family information could be revealed overtime [4].

We also found a significant proportion of participants that reported an increased risk of cancer where no clinically actionable genomic variants were detected. It is possible that the participant sequenced did not inherit the familial disease-causing variant. Given that we assessed the presence of monogenic conditions with the majority being autosomal dominant, we would expect less than 50% (36–37 participants) would carry a clinically relevant variant if adjusted for reduced penetrance. We detected clinically actionable variants in 13.7% of the increased FH cohort; however, it is likely that there are variants of unknown significance present in these participants which over time could be identified as disease-causing. Further possible genetic explanations for the remaining 36.4% of participants in the increased FH risk cohort where no clinically actionable variant was detected include the presence of copy number variants which were not analysed as part of this study, the involvement of genes outside our customised gene panel, or a combination of genetic factors contributing to oligogenic or polygenic inheritance [45]. Testing affected individuals in these families may increase the detection rate further. Health is understood to be influenced by multiple factors including social circumstances, environmental exposures, behavioural patterns, and healthcare systems, with genetic predisposition only contributing 30% [46]. Expanding beyond the focus of monogenic disease risk, family history reflects the contribution of shared hereditary, environmental, and behavioural factors that are present within families [1, 47, 48] which would not be captured by genomic analysis alone. For example, a systematic literature review demonstrated that colorectal cancer risk can increase by twofold if a first-degree family member is affected and increases further with multiple affected family members and/or a diagnosis at a younger age [49], and this is acknowledged in clinical cancer screening guidelines [50]. In our study, participants would still meet cancer surveillance recommendations based on their family history which would not have been evident if genomic sequencing was initiated as a health screen without evaluation of family history as well.

There was one participant in the increased FH risk cohort that was found to carry a LP variant in *AXIN2* and had a family history of breast cancer*. AXIN2* has more recently been described to be associated with colorectal cancer [51], and therefore, the association with breast cancer is not well understood. However, even with the removal of *AXIN2* from the increased FH risk cohort, there is still a fivefold increase (RR 5.8, 95% CI, 2.6–12.4, *p* < 0.0001) in detecting clinically actionable variants in participants with an increased risk family history. Over time, we may learn that this variant is unrelated to the family history or, instead, that it corresponds to the expansion of currently understood genotype-phenotype correlations. Overall, the number of VUS in this study could be considered higher and this is consistent with another study [52] that compared the proportion of VUS variants amongst individuals with a negative personal history of cancer for hereditary breast and ovarian cancer syndrome and Lynch syndrome genes. They found that the VUS rate amongst Asians was higher compared to a European cohort (13.1% vs 5%). Our VUS rate when selecting for the same genes is 10.9%, a comparable number. These findings further support that ethnic disparities exist in the detection of VUS variants and that diversity in clinical variant databases is acutely needed.

Our study was limited by the relatively small number of families found to have a significant family history risk of cancer in comparison to the average risk cohort and the FH not available cohort. The assessment of family history relies on the accuracy of the information provided. Even though there are studies demonstrating family history recollection is reliable [53, 54], as this information is self-reported, it could be incomplete or imprecise, thus impacting the reliability of risk assessment. The assessment of condition-specific risk using established guidelines can be time-consuming and challenging if family history information is incomplete. Further work could involve modifying the risk assessment criteria to optimise how much family information is required as triage for the assessment of pathogenic variants. As cost effectiveness was not explored in this study, further analysis could also include a comparison of cost between the collection of comprehensive family history and the cost of genomic testing.

## Conclusions

This study, to our knowledge, is the first to analyse high quality family history and genomic sequencing data and contributes to current understandings regarding health risks. Our findings show that there is strong concordance between systematic family history collection and presence of clinically actionable variants. In a clinical setting, these findings provide a practical tool to frame the likelihood of detecting a clinically significant variant, manage expectations, and assist with decision-making when genomic sequencing is offered. Despite the reduction in sequencing costs, a strategy using family history to guide selection of individuals for genomic sequencing appears to be financially prudent, particularly in a resource constrained environment. Our findings indicate that family history can assess for personal disease risk beyond genetic factors as evidenced by participants with a family history, yet no concerning genetic variant found. A further understanding of discordant cases can provide crucial indications, such as the presence of novel variants or the data needed to further define variants of unknown significant (when FH risk is increased and there are no clinically actionable variants detected) or the possibility of protective gene-gene and/or gene-environment interactions (when FH risk is average and clinically actionable variants are detected). In conclusion, we have demonstrated that comprehensive family history collection continues to have a significant role in this genomic era.

## Supplementary Information

**Additional file 1: Table S1.** Cancer gene panel. **Fig. S1.** Variant curation and classification flowchart. **Fig. S2.** Cancer prevalence amongst the 73 increased FH risk participants. **Table S2.** Overview of LP/P variants found in the FH not available and FH available groups. **Table S3.** LP/P variants found in the FH not available group**. Table S4.** Clinically actionable variants and associated family history in the increased FH risk cohort. **Table S5.** Variants of unknown significance and associated family history in the increased FH cohort.

## Data Availability

Variant and family history data are available in the main manuscript and its additional supporting files. Whilst consent was obtained from research participants for anonymous use of their data for research purposes, explicit written informed consent to share data through a repository was not obtained. Due to institution policies, the raw genomic data is subject to availability on review by the institution Data Access Committee. Requests for access can be made to the corresponding authors.

## Abbreviations

FH: Family history
VUS: Variants of uncertain significance
ACMG: American College of Medical Genomics
RR: Relative risk
CI: Confidence interval
MAF: Minor allele frequency
