# Correction to: Family history assessment significantly enhances delivery of precision medicine in the genomics era

**DOI:** 10.1186/s13073-021-00916-9

**Published:** 2021-07-05

**Authors:** Yasmin Bylstra, Weng Khong Lim, Sylvia Kam, Koei Wan Tham, R. Ryanne Wu, Jing Xian Teo, Sonia Davila, Jyn Ling Kuan, Sock Hoai Chan, Nicolas Bertin, Cheng Xi Yang, Steve Rozen, Bin Tean Teh, Khung Keong Yeo, Stuart Alexander Cook, Saumya Shekhar Jamuar, Geoffrey S. Ginsburg, Lori A. Orlando, Patrick Tan

**Affiliations:** 1 SingHealth Duke-NUS Institute of Precision Medicine, Singapore Health Services, Singapore, Singapore; 2 Cancer and Stem Cell Biology, Duke-NUS Medical School, Singapore, Singapore; 3 SingHealth Duke-NUS Genomic Medicine Center, Singapore Health Services, Singapore, Singapore; 4 Department of Paediatrics, KK Women’s and Children’s Hospital, Singapore, Singapore; 5 Department of Physiology, National University of Singapore, Singapore, Singapore; 6 Center for Applied Genomics and Precision Medicine, Department of Medicine, Duke University School of Medicine, Durham, NC USA; 7 Cardiovascular and Metabolic Disorders, Duke-NUS Medical School, Singapore, Singapore; 8 Cancer Genetics Service, Division of Medical Oncology, National Cancer Centre Singapore, Singapore, Singapore; 9 Centre for Big Data and Integrative Genomics, Genome Institute of Singapore, Agency for Science Technology and Research, Singapore, Singapore; 10 National Heart Research Institute Singapore, National Heart Centre Singapore, Singapore, Singapore; 11 National Cancer Centre Singapore, Singapore, Singapore; 12 Department of Cardiology, National Heart Centre Singapore, Singapore, Singapore; 13 Paediatric Academic Clinical Programme, Duke-NUS Medical School, Singapore, Singapore; 14 Genome Institute of Singapore, Agency for Science Technology and Research, Singapore, Singapore

**Correction to: Genome Medicine (2021) 13:3.**

**http://orcid.org/10.1186/s13073-020-00819-1**

It was highlighted that in the original article [1] the Funding section was incomplete. This Correction article shows the complete Funding section. The original article has been updated.
