# Supplementary material for: Family history assessment significantly enhances delivery of precision medicine in the genomics era
Source: Genome Med. 2021 Jan 7;13:3. doi: 10.1186/s13073-020-00819-1 (PMC7791763; doi:10.1186/s13073-020-00819-1)
Supplement: Supplementary file 1 — Additional file 1: Table S1. Cancer gene panel. Fig. S1. Variant curation and classification flowchart. Fig. S2. Cancer prevalence amongst the 73 increased FH risk participants. Table S2. Overview of LP/P variants found in the FH not available and FH available groups. Table S3. LP/P variants found in the FH not available group. Table S4. Clinically actionable variants and associated family history in the increased FH risk cohort. Table S5. Variants of unknown significance and associated family history in the increased FH cohort. [file 13073_2020_819_MOESM1_ESM.docx]

**Additional file 1**

**Table S1.** Cancer gene panel

| Gene | OMIM entry | Associated Germline Disorder according to OMIM* and ClinGen^#^ |
| --- | --- | --- |
| AKT1 | 164730 | Cowden syndrome 6 (AD) |
| ALK | 105590 | Neuroblastoma, susceptibility to, 3 (AD) |
| APC | **611731** | **Brain tumor-polyposis syndrome (AD)* Desmoid disease, hereditary* Familial adenomatous polyposis coli/ attenuated familial adenomatous polyposis coli (AD)  Gardner syndrome (AD)*** |
| ATM | 607585 | Ataxia-telangiectasia (AR) Familial ovarian cancer (AD) - limited evidence^#^ Hereditary nonpolyposis colorectal cancer (AD) - moderate evidence^#^ |
| AXIN2 | 604025 | Oligodontia-colorectal cancer syndrome (AD) |
| BAP1 | 603089 | Tumor predisposition syndrome (AD) |
| BARD1 | 601593 | Familial ovarian cancer (AD) - limited evidence^#^ Hereditary breast carcinoma (AD)  Hereditary nonpolyposis colon cancer (AD) - limited evidence^#^ |
| BLM | 210900 | Bloom syndrome (AR) Intestinal cancer (AD) |
| BMPR1A | **601299** | **Juvenile polyposis syndrome, infantile form (AD) Polyposis syndrome, hereditary mixed, 2* Polyposis, juvenile intestinal (AD)*** |
| BRCA1 | **113705** | **Breast-ovarian cancer, familial, 1 (AD) Fanconi anemia, complementation group S (AR) Pancreatic cancer, susceptibility to (AD)*** |
| BRCA2 | **600185** | **Breast-ovarian cancer, familial, 2 (AD) Fanconi anemia (AR) Glioblastoma 3 (AR)* Medulloblastoma (AR, AD)* Pancreatic cancer 2* Prostate cancer (AD)* Wilms tumor (AD)** |
| BRIP1 | 605882 | Fanconi anemia, complementation group (AR) Familial ovarian cancer (AD) |
| BUB1B | 602860 | Mosaic variegated aneuploidy syndrome 1 (AR) Premature chromatid separation trait (AD)* |
| CDC73 | 607393 | Hyperparathyroidism, familial primary (AD)* Hyperparathyroidism-jaw tumor syndrome (AD) Parathyroid adenoma with cystic changes (AD)* Parathyroid carcinoma* |
| CDH1 | 192090 | Blepharocheilodontic syndrome 1 (AD)* Hereditary diffuse gastric adenocarcinoma (AD) Hereditary breast carcinoma (AD) Hereditary nonpolyposis colon cancer (AD)^#^ - limited evidence  Prostate cancer, susceptibility to (AD)* |
| CDK4 | 123829 | Melanoma, cutaneous malignant, 3 (AD) |
| CDKN1B | 600778 | Multiple endocrine neoplasia, type IV (AD) Hereditary nonpolyposis colon cancer (AD)# - limted evidence |
| CDKN1C | 600856 | Beckwith-Wiedemann syndrome (AD) IMAGE syndrome (AD)* |
| CDKN2A | 600160 | Melanoma - pancreatic cancer syndrome (AD) Melanoma and neural system tumor syndrome (AD)* Melanoma, cutaneous malignant, 2 (AD)* Oropharyngeal cancer, multiple* |
| CEBPA | 116897 | Acute myeloid leukemia (AD) |
| CEP57 | 607951 | Mosaic variegated aneuploidy syndrome 2 (AR) |
| CHEK2 | 604373 | Hereditary breast carcinoma (AD) Hereditary nonpolyposis colon cancer (AD)^#^ - limited evidence Li-Fraumeni syndrome (AD)* Prostate cancer, familial, susceptibility to (AD)* |
| DICER1 | 606241 | Familial multinodular goiter (AD) Pleuropulmonary blastoma (AD) Rhabdomyosarcoma, embryonal, 2* |
| DIS3L2 | 614184 | Perlman syndrome (AR) |
| EGFR | 131550 | Non-small cell lung cancer (AD) Inflammatory skin and bowel disease, neonatal, 2 (AR)* |
| EPCAM | 185535 | Colorectal cancer, hereditary nonpolyposis, type 8 (AD) Diarrhea 5, with tufting enteropathy, congenital (AR)* |
| EZH2 | 601573 | Weaver syndrome (AD)* |
| FANCA | 607139 | Fanconi anemia, complementation group A (AR) |
| FANCC | 613899 | Fanconi anemia, complementation group C (AR) |
| FH | 136850 | Hereditary leiomyomatosis and renal cell cancer (AD) Fumarase deficiency (AR)* |
| FLCN | 607273 | Birt-Hogg-Dube syndrome Pneumothorax, primary spontaneous (AD)* |
| GALNT12 | 610290 | Colorectal cancer, susceptibility to, 1 (AD) - limited evidence |
| GATA2 | 137295 | Leukemia, acute myeloid, susceptibility to (AD)* Emberger syndrome (AD)* GATA2 deficiency with susceptibility to MDS/AML (AD) Myelodysplastic syndrome, susceptibility to (AD)* |
| GPC3 | 300037 | Simpson-Golabi-Behmel syndrome, type 1 (XL-R) |
| HOXB13 | 604607 | Prostate cancer, hereditary, 9 (AD)^#^ |
| HRAS | 190020 | Costello syndrome (AD)* Congenital myopathy with excess of muscle spindles (AD)* |
| KIF1B | 605995 | Pheochromocytoma (AD)* Neuroblastoma, susceptibility to, 1 (AD)* Charcot-Marie-Tooth disease, type 2A1 (AD)* Goldberg-Shprintzen megacolon syndrome (AR)^#^ |
| KIT | 164920 | Gastrointestinal stromal tumor (AD) Leukemia, acute myeloid (AD)* Mastocytosis, cutaneous (AD)* Piebaldism (AD)* |
| LZTR1 | 600574 | Schwannomatosis-2, susceptibility to (AD)* Noonan syndrome 10 (AD)^#^ Noonan syndrome 2 (AR)^#^ - limited evidence |
| MAX | 154950 | Pheochromocytoma (AD) |
| MEN1 | **131100** | **Multiple endocrine neoplasia 1 (AD)** |
| MET | 164860 | Papillary renal cell carcinoma (AD) Nonsyndromic genetic deafness (AR) Osteofibrous dysplasia, susceptibility to (AD)* |
| MITF | 156845 | Melanoma, cutaneous malignant, susceptibility to, 8* Waardenburg syndrome type 2 (AD) Waardenburg syndrome, type 2A (AD) COMMAD syndrome (AR)( Tietz albinism-deafness syndrome (AD)* |
| MLH1 | **120436** | **Colorectal cancer, hereditary nonpolyposis, type 2 (AD) Mismatch repair cancer syndrome (AR) Muir-Torre syndrome (AD)*** |
| MLH3 | 604395 | Hereditary nonpolyposis colon cancer (AD) Endometrial cancer, susceptibility to* |
| MRE11A | 600814 | Ataxia-telangiectasia-like disorder 1 (AR)* |
| MSH2 | **609309** | **Colorectal cancer, hereditary nonpolyposis, type 1 (AD) Mismatch repair cancer syndrome (AR) Muir-Torre syndrome (AD)*** |
| MSH3 | 600887 | MSH3-related attenuated familial adenomatous polyposis 4 (AR) Hereditary nonpolyposis colon cancer (AD) - limited evidence |
| MSH6 | **600678** | **Colorectal cancer, hereditary nonpolyposis, type 5 (AD) Mismatch repair cancer syndrome (AR) Endometrial cancer, familial*** |
| MUTYH | **604933** | **MUYTH-related attentuated familial adenomatous polyposis (AR) Familial ovarian cancer (AD)^#^ - limited evidence** |
| NBN | 602667 | Nijmegen breakage syndrome (AR)  Leukemia, acute lymphoblastic* Hereditary breast carcinoma (AD)^#^ - limited evidence Aplastic anemia* |
| NF1 | 162200 | Neurofibromatosis, type 1 (AD) |
| NF2 | **607379** | **Neurofibromatosis, type 2 (AD)** |
| NKX2-1 | 600635 | Thyroid cancer, nonmedullary, 1 (AD)* Chorea, hereditary benign (AD)* Choreoathetosis, hypothyroidism, and neonatal respiratory distress (AD)* |
| NTHL1 | 602656 | Familial adenomatous polyposis 3 (AR)* |
| PALB2 | 610355 | Familial ovarian cancer (AD) Hereditary breast carcinoma (AD) Fanconi anemia, complementation group N (AR) Hereditary nonpolyposis colon cancer (AD)^#^ - limited evidence Pancreatic cancer, susceptibility to, 3* |
| PALLD | 608092 | Pancreatic cancer, susceptibility to, 1* |
| PDGFRA | 173490 | Gastrointestinal stromal tumor (AD) |
| PHOX2B | 603851 | Neuroblastoma, susceptibility to, 2 (AD)* Central hypoventilation syndrome, congenital, with or without Hirschsprung disease 209880 (AD)* |
| PIK3CA | 171834 | Cowden syndrome 5 (AD)* |
| PMS2 | **600259** | **Colorectal cancer, hereditary nonpolyposis, type 4 (AR) Mismatch repair cancer syndrome (AR)** |
| POLD1 | 174761 | Polymerase proofreading-related adenomatous polyposis (AD)^#^ Colorectal cancer, susceptibility to, 10 (AD) Mandibular hypoplasia, deafness, progeroid features, and lipodystrophy syndrome (AD)* |
| POLE | 174762 | Polymerase proofreading-related adenomatous polyposis (AD)# Colorectal cancer, susceptibility to, 10 (AD)  FILS syndrome (AR)* IMAGE-I Syndrome* |
| POT1 | 606478 | Melanoma, cutaneous malignant, susceptibility to, 10 (AD) Glioma susceptibility 9 (AD) |
| PRKAR1A | 188830 | Carney complex, type 1 (AD)  Acrodysostosis 1, with or without hormone resistance (AD)* Myxoma, intracardiac (AD)* Pigmented nodular adrenocortical disease, primary, 1 (AD)* |
| PTCH1 | 601309 | Nervoid basal cell carcinoma syndrome (AD) Holoprosencephaly (AD)* |
| PTEN | **601728** | Cowden syndrome 1 (AD) Lhermitte-Duclos syndrome (AD)* Macrocephaly/autism syndrome (AD)* VATER association with macrocephaly and ventriculomegaly (AR)* Glioma susceptibility 2* Meningioma (AD)* |
| RAD50 | 604040 | Nijmegen breakage syndrome-like disorder (AD)* Hereditary breast carcinoma^#^ - limited evidence |
| RAD51C | 602774 | Familial ovarian cancer (AD) Fanconi anemia, complementation group O (AR) |
| RAD51D | 602954 | Familial ovarian cancer (AD) |
| RB1 | **614041** | **Retinoblastoma (AD)** |
| RET | **164761** | **Familial medullary thyroid carcinoma (AD) Multiple endocrine neoplasia IIA (AD) Multiple endocrine neoplasia IIB (AD) Hirschsprung disease, susceptibility to,1, (AD) Pheochromocytoma (AD)* Central hypoventilation syndrome, congenital (AD)*** |
| RHBDF2 | 614404 | Palmoplantar keratoderma-esophageal carcinoma syndrome (AD) |
| RUNX1 | 151385 | Hereditary thrombocytopenia with normal platelets-hematological cancer predisposition syndrome (AD) Leukemia, acute myeloid (AD)* |
| SDHA | 600857 | Paragangliomas 5 (AD) Leigh syndrome (Mi, AR) Mitochondrial respiratory chain complex II deficiency (AR)* |
| SDHAF2 | **613019** | **Paragangliomas 2 (AD)** |
| SDHB | **185470** | **Pheochromocytoma (AD) Paragangliomas 4 (AD) Gastrointestinal stromal tumor (AD)*** |
| SDHC | **602413** | **Paragangliomas 3 (AD) Gastrointestinal stromal tumor (AD)*** |
| SDHD | **602690** | **Pheochromocytoma (AD) Paragangliomas 1, with or without deafness (AD) Mitochondrial complex II deficiency (AR)* Paraganglioma and gastric stromal sarcoma*** |
| SLX4 | 613278 | Fanconi anemia, complementation group P (AR) |
| SMAD4 | **600993** | **Juvenile polyposis/hereditary hemorrhagic telangiectasia syndrome (AD) Myhre syndrome (AD)*** |
| SMARCA4 | 603254 | Coffin-Siris syndrome 4 (AD) Rhabdoid tumor predisposition syndrome 2 (AD) |
| SMARCB1 | 601607 | Rhabdoid tumor predisposition syndrome 1 (AD) Coffin-Siris syndrome 3 (AD)* Schwannomatosis-1, susceptibility to (AD)* |
| SMARCE1 | 603111 | Familial meningioma, (AD) Coffin-Siris syndrome 5 (AD)* |
| STK11 | **602216** | **Peutz-Jeghers syndrome (AD)** |
| SUFU | 607035 | Medulloblastoma (AD) Basal cell nevus syndrome (AD)* Joubert syndrome 32 (AR)* Meningioma, familial, susceptibility to (AD)* |
| TERT | 187270 | Dyskeratosis congenita, autosomal dominant 2 (AD) Dyskeratosis congenita, autosomal recessive 4 (AR)* Leukemia, acute myeloid (AD)* Melanoma, cutaneous malignant, 9 (AD)* Pulmonary fibrosis and/or bone marrow failure, telomere-related, 1 (AD)* |
| TMEM127 | 613403 | Pheochromocytoma, susceptibility to (AD) |
| TP53 | **191170** | **Bone marrow failure syndrome 5 (AD) Li-Fraumeni syndrome (AD) Adrenocortical carcinoma, pediatric (AD)* Basal cell carcinoma 7 (AD)* Choroid plexus papilloma (AD)* Colorectal cancer (AD)* Glioma susceptibility 1 (AD)* Osteosarcoma (AR)*** |
| TSC1 | **605284** | **Tuberous sclerosis-1 (AD)** |
| TSC2 | **191092** | **Tuberous sclerosis-2 (AD)** |
| TSHR | **603372** | **Thyroid carcinoma with thyrotoxicosis (AD)*  Hyperthyroidism, familial gestational (AD)*  Hyperthyroidism, nonautoimmune (AD)*** |
| VHL | **608537** | **von Hippel-Lindau syndrome (AD) Pheochromocytoma (AD)* Erythrocytosis, familial, 2 (AR)*** |
| WT1 | **607102** | **Wilms tumor, type 1 (AD) Denys-Drash syndrome (AD)* Frasier syndrome (AD)* Meacham syndrome* Nephrotic syndrome, type 4 (AD)*** |
| XRCC2 | 600375 | Fanconi anemia, complementation group U (AR)* Breast cancer (AD) Limited evidence^#^ |

Genes bolded are in the ACMG 59 secondary findings gene list (ACMG SF v2.0)

Genes underlined are associated with autosomal recessive inheritance

*entry in OMIM only

^#^ entry in ClinGen only

**
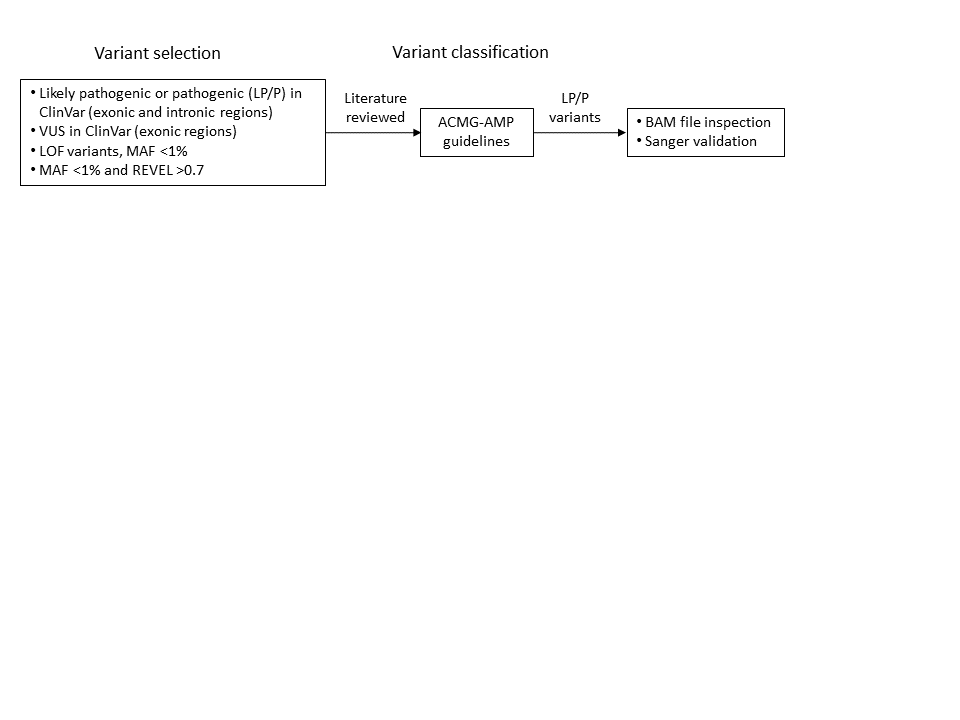
**

**Fig. S1.** Variant curation and classification flowchart

**Fig. S2.** Cancer prevalence amongst the 73 increased FH risk participants

**Table S2.** Overview of LP/P variants found in the FH not available and FH available groups

|  | **Total population n=1750** | | | | |
| --- | --- | --- | --- | --- | --- |
|  | FH not available n=884 |  | | FH available n=866 | |
| **Gene** |  | | Total n=866 | Average FH risk n=793 | Increased FH risk n=73 |
| *ATM* | 1 | | 4 | 2 | 2 |
| *AXIN2* | - | | 1 | - | 1 |
| *BLM* | - | | 1 | 1 | - |
| ***BRCA1*** | - | | 2 | - | **2** |
| ***BRCA2*** | **2** | | **5** | **3** | **2** |
| *BRIP1* | 2 | | 1 | 1 | - |
| *CDKN1B* | 1 | | - | - | - |
| *CDKN2A* | 1 | | - | - | - |
| *DICER1* | - | | 1 | 1 | - |
| *GPC3* | - | | 1 | 1 | - |
| *LZTR1* | - | | 2 | 2 | - |
| *MAX* | 1 | | - | - | - |
| ***MSH2*** | - | | **1** | - | **1** |
| ***MSH6*** | **1** | | - | - | - |
| *NF1* | 1 | | - | - | - |
| *RAD50* | 2 | | 2 | 1 | 1 |
| *RAD51C* | 2 | | 1 | 1 | - |
| *RAD51D* | 1 | | 3 | 3 | - |
| *SDHA* | 1 | | - | - | - |
| *SUFU* | - | | 1 | - | 1 |
| ***TP53*** | **1** | | - | - | - |
| *XRCC2* | - | | 1 | 1 | - |
| **Cancer gene panel** |  | |  |  |  |
| Carriers total % | 17 (1.9) | | 27 (3.1) | 17 (2.1) | 10 (13.7) |
| **ACMG cancer genes** |  | |  |  |  |
| Carriers total % | 4 (0.5) | | 8 (0.9) | 3 (0.4) | 5 (6.8) |

Genes bolded are in the ACMG 59 secondary finding gene list (ACMG SF v2.0)

**Table S3.** LP/P variants found in the FH not available group

| **Family ID** | **Gene variant** | **Family ID** | **Gene variant** |
| --- | --- | --- | --- |
| **1672** | *ATM* c. 6115 G >A p.E2039K | **399** | *MSH6* c.1998dupT p.S666fs |
| **878** | *BRCA2* c. 3109 C >T p.Q1037X | **1618** | *NF1* c.3277G>A p.V1093M |
| **1723** | *BRCA2* c.4245delG p.E1415fs | **153** | *RAD50* c.2263C>T p.Q755X |
| **616** | *BRIP1* c.2392C>T p.R798X | **1299** | *RAD50* c.2263C>T p.Q755X |
| **167** | *BRIP1* c.3072delG p.G1024fs | **151, 521** | *RAD51C* c.905-2A>C |
| **1537** | *CDKN1B* c.487_491del p.Q163fs | **1425** | *RAD51D* c.898C>T p.R300X |
| **549** | *CDKN2A* c.159G>A p.M53I | **915** | *SDHA* c.A1G:p.M1V |
| **566** | *MAX* c.154delC p.L52fs | **661** | *TP53* c.731G>A p.G244D |

transcripts: ATM: NM_000051.3, BRCA2: NM_000059.3, BRIP1:NM_032043..2, CDKN1B:NM_004064.4, CDKN2A:NM_000077.4, MAX:NM_001271068.1, MSH6:NM_000179.3, NF1:NM_000267.3, RAD50:NM_005732.4, RAD51C: NM_058216.3, RAD51D: NM_002878.3,: SDHA:NM_004168.4, TP53:NM_000546.5

**Table S4.** Clinically actionable variants and associated family history in the increased FH risk cohort

| Family ID | Gene variant | Family history |
| --- | --- | --- |
| Increased FH risk | |  |
| 8 | *ATM* c.8224_8225delAA p.Asn2742HisfsTer4 | Paternal uncle dx colorectal cancer, another paternal uncle dx lung cancer, paternal grandmother dx colorectal cancer |
| 9 | *ATM*  c.7702A>T p.R2568X | Sister dx breast cancer 50s, maternal uncle dx stomach cancer 40s d.40s, paternal aunt dx unknown cancer d.75y, paternal grandfather dx unknown cancer d.50s |
| 2 | *AXIN2* c.1614_1642del p.R538fs | Father dx heart attack 47y, bypass 48y, heart attack 50y and d. heart failure 65y, mother dx Parkinson 64y, maternal grandmother dx esophageal cancer d.70y, maternal grandfather dx lymphatic cancer d.71y, maternal aunt 1 dx breast cancer 45y, maternal aunt 2 dx nipple cancer 40y, paternal aunt 2 dx breast cancer d.45y, paternal uncle dx mild stroke 50y |
| 11 | *BRCA1* c. 4435C >T p.Q1479X | Mother dx ovarian cancer 60s, father dx prostate cancer 50y |
| 37 | *BRCA1* c.2585dupA p.N863fs | Sister dx stomach cancer d.37y, another sister dx ovarian cancer, maternal aunt dx unknown cancer d.65y |
| 54 | *BRCA2* c.4338delT p.I1446fs | Maternal aunt dx ovarian cancer 60s and adenomas, maternal grandmother dx adenomas |
| 79 | *BRCA2* c.7379_7382del p.K2460fs | Mother dx unknown cancer 40s, sister dx breast cancer 40s |
| 35 | *MSH2* c. 1102G>T p.E368X | Father dx colorectal cancer 30s, pancreatic cancer 60s, sister dx ovarian cancer 20s, uterine cancer 30s, paternal grandmother dx liver cancer 40s |
| 58 | *RAD50* c.2165dupA p.L723fs | Father dx liver cancer 40s d.40s, mother dx thyroid disorder, maternal grandmother dx lung cancer 20s d.20s, maternal grandfather dx unknown cancer 40s d.40s, paternal grandmother dx breast cancer 50s d.50s |
| 6 | *SUFU* c.71dupC p.A25fs | Daughter dx brain cancer in infancy |

dx:diagnosis, d.: died, y:year

transcripts: ATM: NM_000051.3, AXIN: NM_004655.4, BRCA1:NM_007300.4, BRCA2: NM_000059.3, MSH2: NM_000215.3, RAD50: NM_005732.4, SUFU: NM_016169.4

**Table S5.** Variants of unknown significance and associated family history in the increased FH cohort

| Family ID | Gene variant | Family history |
| --- | --- | --- |
| 39 | *BRCA1* c. 2006T>C p.M669T | Mother dx breast cancer 30's and adenoma, maternal aunt dx colorectal cancer 50s. |
| 28 | *BRCA1* c.4927A>C p.K1643Q | Father dx high cholesterol and hypertension 40s, mother dx high cholesterol and hypertension 40s, brother dx diabetes 20s and prostate cancer 30s, maternal grandmother dx heart disease 70s, paternal grandmother dx diabetes 50s d.80s |
| 29 | *BRCA1* c.5278G>C p.D1760H | Mother dx breast cancer dx unknown, d.50's, maternal aunt ovarian cancer in 60's and breast cancer dx unknown |
| 12 | *BRCA1* c.397C>T p.R133C | Father dx hypertension 59y, mother dx thyroid issues late 50s and hypertension early 60s, maternal aunt 1 dx breast cancer late 40s, maternal uncle 1 dx nose cancer 40s, 2 maternal aunts and 1 maternal uncle dx stroke 50s, paternal grandfather dx throat cancer d.50s, paternal uncle dx colorectal cancer 60s |
| 769 | *BRCA1* c. 2286 A>T p.R762S | Sister dx stomach ca d.43y, father dx lung ca d.68y, mother dx high cholesterol 70s, paternal grandmother dx breast ca d.60s, paternal uncle 1 dx lung ca d.60s, paternal uncle 2 dx colorectal cancer 60s, paternal uncle 3 dx unknown ca 60s, maternal uncle dx lung ca d.65y (no paternal aunts) |
| 717 | *MLH1* c. 86 C>A p.A29D | Maternal uncle 3 dx colorectal cancer 50s, maternal aunt 3 d. lung disease 40s, maternal cousin dx lupus adolescence d.20s, paternal grandmother diagnosed dx ovarian ca 70s d.70s, paternal uncle 3 dx lung ca 50s d.50s |
| 682 | *MSH3* c. 886 C>T p.R296C | Mother dx ovarian ca 50s and hypertension 60s, father dx hypertension 70s, 2 sisters dx ovarian cysts, maternal aunt dx breast ca 40s, paternal aunt d.19y sudden cardiac death, 2 paternal uncles dx Parkinson |
| 9 | *MSH3* c. 886 C>T p.R296C | Sister dx breast cancer 50s, maternal uncle dx stomach cancer 40s d.40s, paternal aunt dx unknown cancer d.75y, paternal grandfather dx unknown cancer d.50s |
| 49 | *PMS2* c.2437C>T p.R813W | Maternal grandmother dx colorectal cancer 60s d.70s, maternal grandfather dx nose cancer 50s d.50s, maternal aunt 1 dx colorectal and liver cancer, maternal uncle 1 dx lung cancer, maternal uncle 2 dx lung cancer and liver failure, maternal uncle 3 dx lung cancer, maternal uncle 4 dx hypertension and diabetes, paternal aunt 5 dx hypertension and paternal uncle 4 dx hypertension |

dx:diagnosis, d.: died, y:year

transcripts: BRCA1:NM_007300.4, MLH1:NM_000249.4, MSH3:NM_002439.5, PMS2:NM_000535.7
